# Retrospective analysis of the hemodynamic effects of induction of barbiturate coma in patients with refractory elevated intracranial pressure

**DOI:** 10.1186/cc12268

**Published:** 2013-03-19

**Authors:** S D'Hollander, M Vander Laenen, JB Gillet, C De Deyne, R Heylen, W Boer, F Jans

**Affiliations:** 1 ZOL Genk, Belgium; 2 UZ Leuven, Belgium

## Introduction

Standard clinical practice in patients with elevated intracranial pressure (ICP) is to keep cerebral perfusion pressure (CPP) at (or above) 60 mmHg: we performed this retrospective study to analyze whether induction of barbiturate coma impedes compliance to this target CPP value. It has been repetitively shown that, although proven as an efficient therapy for refractory elevated ICP, barbiturate coma may cause a decrease in mean arterial pressure (MAP) and CPP [1].

## Methods

All patients that had received sodium thiopental in our ICU during 2011 were identified and their medical and nursing records were retrospectively analyzed. The effect of administration of sodium thiopental (loading dose 3 to 5 mg/kg, followed by a continuous infusion adjusted to obtain an EEG burst suppression ratio between 40 and 80%) on MAP, ICP and CPP was evaluated. Values are reported as mean ± SD. The changes over time in ICP, MAP and CPP are reported as means with their 95% CI.

## Results

In 2011, a total of 20 patients were treated in our ICU with barbiturate coma for refractory elevated ICP. In five patients, systemic hypothermia was simultaneously induced. These patients were excluded from further analysis. Six patients died during their stay in the ICU and nine patients could be discharged to the neurosurgical ward. The mean peak ICP value before induction of barbiturate coma was 26 ± 3 mmHg, and the ICP value 6 hours later was 20 ± 6 mmHg. The MAP at those same time points was 91 ± 12 mmHg and 81 ± 11 mmHg, respectively. The CPP, calculated from both previous values, was 66 ± 13 mmHg and 61 ± 11 mmHg, respectively. At both time points, the dose of noradrenaline was comparable (10.1 ± 9.6 μg/minute and 11.5 ± 13.2 μg/minute, respectively). Analysis of the change in ICP, MAP and CPP over the 6-hour period showed a significant decrease in ICP of -5.8 mmHg (-9.3; -2.3) (mean, 95% CI). The MAP significantly decreased by -10.4 mmHg (-19.1; -1.7). The decrease in CPP was not significant (-4.6 mmHg (-14.2; 5.0)).

## Conclusion

This retrospective study indicates that inducing a thiopental coma in patients with refractory elevated ICP effectively reduces ICP. Although the concomitant reduction in MAP was significant, the resulting decrease in CPP was small and nonsignificant. Moreover, the target CPP of 60 mmHg was maintained after induction of barbiturate coma.
